# Heterogeneity of the tumor vasculature: the need for new tumor blood vessel type-specific targets

**DOI:** 10.1007/s10585-012-9500-6

**Published:** 2012-06-13

**Authors:** Janice A. Nagy, Harold F. Dvorak

**Affiliations:** Departments of Pathology, The Center for Vascular Biology Research, Beth Israel Deaconess Medical Center and Harvard Medical School, 330 Brookline Ave, RN227C, Boston, MA 02215 USA

**Keywords:** Angiogenesis, VPF/VEGF-A, Ad-VEGF-A^164^, Aflibercept (VEGF Trap), Rapamycin

## Abstract

Therapies directed against VEGF-A and its receptors are effective in treating many mouse tumors but have been less so in treating human cancer patients. To elucidate the reasons that might be responsible for this difference in response, we investigated the nature of the blood vessels that appear in human and mouse cancers and the tumor “surrogate” blood vessels that develop in immunodeficient mice in response to an adenovirus expressing VEGF-A^164^. Both tumor and tumor surrogate blood vessels are heterogeneous and form by two distinct processes, angiogenesis and arterio-venogenesis. The first new angiogenic blood vessels to form are mother vessels (MV); MV arise from preexisting venules and capillaries and evolve over time into glomeruloid microvascular proliferations (GMP) and subsequently into capillaries and vascular malformations (VM). Arterio-venogenesis results from the remodeling and enlargement of preexisting arteries and veins, leading to the formation of feeder arteries (FA) and draining veins (DV) that supply and drain angiogenic vessels. Of these different blood vessel types, only the two that form first, MV and GMP, were highly responsive to anti-VEGF therapy, whereas “late”-formed capillaries, VM, FA and DV were relatively unresponsive. This finding may explain, at least in part, the relatively poor response of human cancers to anti-VEGF/VEGFR therapies, because human cancers, present for months or years prior to discovery, are expected to contain a large proportion of late-formed blood vessels. The future of anti-vascular cancer therapy may depend on finding new targets on “late” vessels, apart from those associated with the VEGF/VEGFR axis.

It has been known for some time that most tumors need to generate a vascular supply if they are to grow beyond small size. Folkman [1] proposed that tumors induced the formation of new blood vessels by secreting a “tumor angiogenesis factor” or “TAF”. It is now clear that a number of growth factors can induce angiogenesis, and tumors can secrete many of them. However, it is also generally agreed that the most important TAF, at least for initial tumor growth, is vascular permeability factor/vascular endothelial growth factor (VPF/VEGF or, more simply, VEGF-A) [2, 3]. Further, although VEGF-A interacts with a number of different receptors, VEGFR-2 (KDR, flk-1) is the one that is essential for the signaling pathways that promote angiogenesis and vascular permeability [4].

Folkman also proposed that drugs targeting TAFs would be effective in preventing tumor growth [1], and therefore there was great excitement when it was shown that antibodies to VEGF-A inhibited fluid accumulation in the case of ascites tumors [5] and prevented the growth of many solid mouse tumors and tumor xenografts [6, 7]. Based on these animal studies, it was anticipated that therapies directed against VEGF-A or VEGFR-2 would open a new phase of therapy against human cancers. Unfortunately, the promise of this approach has not yet been fulfilled. Bevacizumab (Avastin; Genentech), a humanized monoclonal antibody directed against VEGF-A and the most studied of the anti-VEGF-A/VEGFR drugs, prolongs the life of patients with advanced colon cancer by only 4–5 months and then only when accompanied by chemotherapy [8]. When administered along with chemotherapy, bevacizumab and small molecule receptor tyrosine kinase inhibitors directed against VEGFR-2 are of some benefit in several other cancers; however, although these therapies often prolong progression-free survival by a matter of months, they generally do not extend life [9]. Therefore, they are not the blockbusters that had been hoped for and some have wondered whether they are “boon or bust” [10]. Why have these drugs not been more successful in treating human cancer, given their widely agreed upon effectiveness in mice? A number of reasons have been offered and will be considered here. However, before addressing this important question, we will first consider two other issues that provide insight into the failure of anti-VEGF/VEGFR therapy to work better. These are a description of the types of blood vessels that VEGF-A-secreting tumors generate and the steps and mechanisms by which these blood vessels form.

## Heterogeneity of the tumor vasculature

**Fig. 1:**
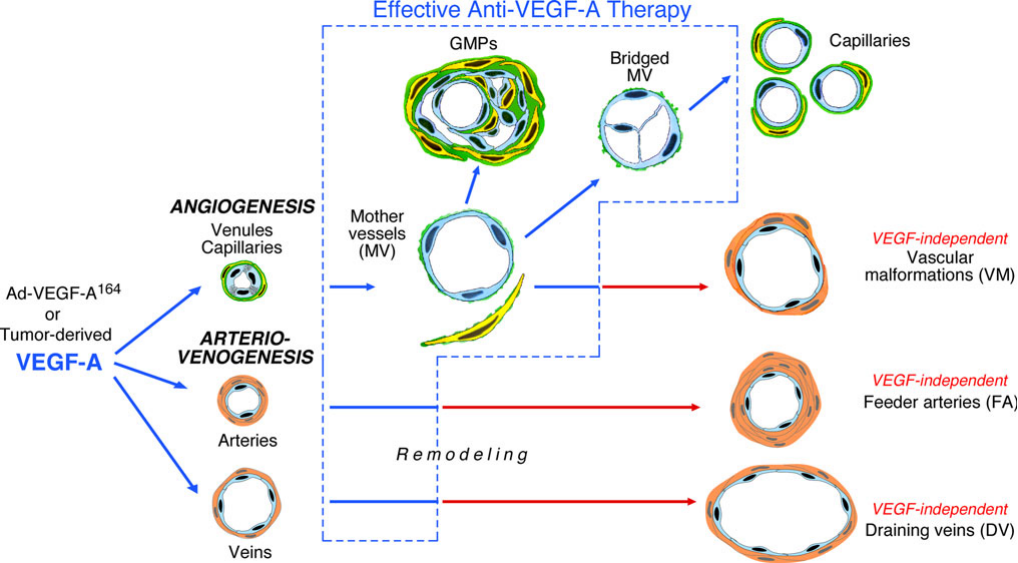
Schematic diagram of the steps by which VEGF-A^164^-induces angiogenesis and arterio-venogenesis. The blood vessels responsive to anti-VEGF-A therapy are enclosed within the *box outlined* with a *dashed line*. (Modified after Fig. 1 in [27])

Warren [11] in the late 1970s, and we and others more recently (reviewed in [12–14]), have analyzed the types of blood vessels that are found in tumors. These studies have shown that the tumor vasculature is highly heterogeneous, and, for the most part, very different from that found in normal tissues. Examining several human cancers, we were able to identify six distinct tumor blood vessel types (Table 1). Further, using an adenovirus engineered to express VEGF-A^164^, we were able to replicate each of these in the form of tumor “surrogate” vessels in immunodeficient mice [14, 15]. This model system allowed us to elucidate the steps and mechanisms by which each vessel type formed initially and evolved over time (Fig. 1). Unlike tumors, which express high levels of VEGF-A over extended periods of time, Ad-VEGF-A^164^ gives rise to a single pulse of VEGF-A^164^. This VEGF-A^164^ pulse is initially of similar magnitude to that found in many tumors. However, unlike in tumors, local VEGF-A^164^ levels fall off dramatically over a matter of weeks because the adenoviral insert carrying the transgene is not integrated into the cellular genome and so is discarded from the cell. In contrast, tumors continue to express VEGF-A at high levels over long periods of time; thus, they continually induce the formation of new blood vessels, while, concurrently, causing previously formed vessels to differentiate into more stable forms. Given this mixture of “early” and “late” vessels, it becomes difficult to follow progression of newly formed vessels as they evolve over time in tumors. In contrast, at Ad-VEGF-A^164^ injection sites, the formation of new blood vessels is restricted temporally and their evolution into late vessels can therefore be followed sequentially. Studies with the Ad-VEGF-A^164^ model demonstrated that four of the six tumor vessel “surrogates” develop by angiogenesis, i.e., they derive initially from preexisting small blood vessels, namely, venules and capillaries. In addition, both VEGF-A-secreting tumors and Ad-VEGF-A^164^ induce abnormal arteriogenesis and venogenesis, i.e., they cause pre-existing arteries and veins to enlarge and remodel and these feed and drain the angiogenic vascular bed. There is every reason to believe that similar processes are going on in tumors.

**Table 1:** Classification of tumor/tumor surrogate blood vessels

Process involved	Vessel type	Vessel properties
Angiogenesis	Mother vessels (MV)	Large, thin-walled, hyperpermeable, lightly fenestrated pericyte-poor sinusoids that are engorged with red blood cells.
	Capillaries	Formed from MV by a process that involves intra-luminal bridging and intussusception.
	Glomeruloid microvascular proliferations (GMP)	Poorly organized vascular structures that resemble renal glomeruli macroscopically. GMP are comprised of endothelial cells and pericytes with minimal vascular lumens and reduplicated basement membranes.
	Vascular malformations (VM)	Mother vessels that have acquired an often asymmetric coat of smooth muscle cells and/or fibrous connective tissue.
Arterio-venogenesis	Feeder arteries (FA)	Enlarged, often tortuous arteries and veins that are derived from preexisting arteries and veins. They extend radially from the tumor mass, supplying and draining the angiogenic vessels within.
	Draining veins (DV)

## Types of tumor and tumor “surrogate” blood vessels and their generation

“Mother” vessels (MV) are the first new type of angiogenic blood vessel to appear, both in tumors and also in response to Ad-VEGF-A^164^ [15, 16] (Fig. 1). MV are greatly enlarged sinusoids that are highly permeable to plasma proteins and to other circulating macromolecules [13, 14]. They begin to develop from preexisting venules and capillaries within hours of injection of tumor cells or of Ad-VEGF-A^164^ into mouse tissues. We predicted that vascular basement membrane (BM) degradation would be an important step in MV development because BM are non-compliant (non-elastic) structures that normally restrict microvessel expansion [17]. Swayne had demonstrated the importance of BM in maintaining microvessel size in studies by demonstrating that progressive increases in intravascular pressure were only able to increase vascular cross-sectional area by ~30 % before vessels burst [18], i.e., far less than the three to five-fold increase in area typical of MV. Testing this hypothesis, we found that over the course of a few days after injecting Ad-VEGF-A^164^ or tumor cells into mouse tissues, BM staining for laminin and type IV collagen, the most abundant components of vascular BM, was progressively lost in developing MV [17]. Further, western blots revealed progressive fragmentation of both proteins. Gene chip studies revealed that cathepsin transcripts were increased locally, and this finding was confirmed and extended by RT-PCR and at the protein level by immunohistochemistry. Further, western blots revealed that activated forms of three cathepsins, B, S, and L, increased substantially as MV developed, and immunohistochemistry selectively localized increased cathepsin activity to the pericytes associated with developing MV. In normal tissues the action of cathepsins is opposed by a family of endogenous inhibitors called cysteine protease inhibitors (CPI). As MV formed, expression of these inhibitors progressively decreased in both endothelial cells and pericytes. Thus, BM degradation was induced in MV by increased expression of cathepsins and decreased expression of CPI, i.e., by an upsetting of the cathepsin/CPI balance that normally maintains BM integrity and so microvascular size. As a consequence of BM degradation, pericytes lost their attachments to endothelial cells, and endothelial cells, no longer restrained by BM or attached pericytes, underwent cellular thinning as their lumens expanded in response to intravascular pressure. Increased lumen size requires an increase in endothelial cell surface area and so an increase in plasma membrane. This was provided, at least in part, by vesiculo-vacuolar organelles (VVOs), clusters of hundreds of interconnected vesicles and vacuoles contained within the cytoplasm of normal venular endothelial cells [19]. VVOs have an important role in the transport of macromolecules across venules in the acute vascular hyperpermeability induced by VEGF-A, histamine, etc. [20, 21]. The membrane stored in VVOs amounts to more than twice that found in the plasma membranes of normal venular endothelial cells. As the formerly cuboidal endothelial cells of normal venules flattened, VVOs fused with the plasma membrane, contributing to the plasma membrane expansion necessary for MV formation.

MV are typically unstable blood vessels as their lack of pericytes, basement membrane support, and sluggish blood flow make them susceptible to thrombosis or collapse. MV are therefore transitional structures that evolve into one or another type of daughter vessel: capillaries, glomeruloid microvascular proliferations (GMP) and vascular malformations (VM) [13, 14] (Fig. 1).

Capillaries form from MV by a process of internal bridging as endothelial cells extend thin, “tip-cell-like” processes into the MV lumen rather than externally as in vascular “sprouting” [13, 14]. These endothelial cell processes grow to form transluminal bridges that divide MVs into smaller, capillary-sized structures that eventually separate from each other by a process of intussusception. GMP result from a proliferation of endothelial cells and pericytes that fill MV lumens and divide them into much smaller channels that are enveloped by redundant layers of BM [15, 22]. Like MV, GMP are hyperpermeable to macromolecules, but, because they are poorly perfused, account for relatively little plasma extravasation. Finally, vascular malformations (VM) derive from MV that have acquired a supporting smooth muscle cell coating. VM are readily distinguished from normal arteries and veins by their inappropriately large size (for their location) and by their thinner and often asymmetric muscular coat. They, and the capillaries that form in parallel, are not hyperpermeable to plasma proteins.

In addition to inducing angiogenesis, tumors and Ad-VEGF-A^164^ also stimulate abnormal arteriogenesis and venogenesis, leading to the formation of the large, often tortuous feeder arteries (FA) and draining veins (DV) that supply and drain the tumor microvasculature [13, 14]. The mechanisms by which these vessels develop have been little investigated. However, once induced to form in response to Ad-VEGF-A^164^, FA and DV, like VM, persisted indefinitely.

## Why doesn’t anti-VEGF-A/VEGFR therapy work better in cancer patients?

A number of reasons have been offered for the disappointing ineffectiveness of anti-VEGF/VEGFR drugs in treating human cancers [23–25]. These include the need for better dosing strategies; the frailty of cancer patients who are much sicker than tumor-bearing mice and therefore cannot withstand the toxicities associated with high dose therapy; and “vascular normalization”, a period of time following anti-VEGF/VEGFR therapy in which tumor vessels normalize structurally and lose their hyperpermeability. An additional important reason is that an impaired vascular supply may not result in the killing of all tumor cells, leaving survivors hypoxic. Hypoxic tumor cells reprogram their transcriptional profile to increase the synthesis of VEGF-A as well as panoply of other growth factors that together can overwhelm anti-angiogenesis therapy.

All of the reasons listed above are valid. However, they seem insufficient to explain fully the lack of anti-VEGF/VEGFR drug effectiveness in cancer patients relative to those in mice, and other possibilities must be considered. Closer inspection of published mouse tumor data provides some clues. The major successes of anti-VEGF-A/VEGFR therapies in mice have been in the prevention of the growth of freshly transplanted mouse tumors and tumor xenografts [23, 26] (for additional references, see [27, 28]). In contrast, when anti-VEGF/VEGFR therapy is delayed and administered to mice that have established autochthonous or transplanted tumors, the benefits are of lesser magnitude [23, 26]. Obviously the difference in treatment regimen timing is important because freshly transplanted tumors do not mimic human cancer; in patients, cancers have been growing for months or years before they are discovered and treated. The better question, then, becomes, why does anti-VEGF-A/VEGFR therapy work better on newly transplanted tumors than on established tumors?

We suggest that the different types of blood vessels that tumors induce are likely to provide important insights. As noted, tumor blood vessels are heterogeneous and form linearly over time by two distinct but interrelated processes, angiogenesis and arterio-venogenesis (Fig. 1). The initial or “early” vessels induced by VEGF-A are MV and actively remodeling arteries and veins; these subsequently evolve over a period of weeks to months into stable “late” vessels, i.e., capillaries, VM, FA and DV. Based on this understanding of tumor vessel development, we postulated that “early” and “late” vessels might differ in their susceptibility to anti-VEGF-A/VEGFR therapy. We offer the hypothesis that “early” vessels are susceptible to anti-VEGF-A/VEGFR therapy whereas “late” vessels, though formed from “early” vessels that are induced by VEGF-A, have lost their VEGF-A dependence and have therefore become resistant to anti-VEGF-A/VEGFR therapy. Established tumors, whether mouse or human, are expected to contain a mixture of “early” and “late” vessels. “Early” vessels would predominate initially; however, as tumors grow over time, the population of “late” vessels would be expected to become proportionately greater.

The hypothesis just put forward has consequences. It predicts that 1. “Late” vessels are less dependent on exogenous VEGF-A than “early” vessels and 2. “Late” vessels are relatively resistant to anti-VEGF-A/VEGFR therapy compared with “early” vessels.

## Testing the hypothesis: the differential sensitivity of “early” and “late” vessels to agents interfering with the VEGF-A/VEGFR axis

If our hypothesis has merit, then “late” vessels should be less dependent on the VEGF/VEGFR axis than “early” vessels. Initial experiments demonstrated that “early” vessels such as MV and GMP stained strongly for VEGFR-2, the VEGF-A receptor responsible for both VEGF-A-induced angiogenesis and increased permeability; in contrast, staining for VEGFR-2 was greatly reduced or largely negative in VM, FA and DV [28]. This finding, consistent with our hypothesis, suggests that VEGFR-2 signaling is less important in “late” than in “early” vessels.

To test our hypothesis more directly, we evaluated the effects of two drugs that act at different stages in VEGF-A signaling [28, 29]. One such drug, rapamycin, inhibits mTOR, a downstream target of the VEGF-A-Akt pathway. When administered before and coincident with Ad-VEGF-A^164^ injection into tissues, rapamycin effectively inhibited local vascular Akt and S-6 phosphorylation and prevented the formation of MV and the vascular hyperpermeability and edema that accompany their formation [29]. When administered slightly later, at a time when MV had already formed, differentiation of MV into GMP and VM was greatly reduced. However, when administered still later, when VM, FA and DV predominated, rapamycin had little effect.

Very recently we obtained similar results with a second drug, Aflibercept (VEGF-Trap). Aflibercept is a human soluble decoy receptor protein with high affinity for all of the VEGF-A isoforms, as well as for VEGF-B and placental growth factor [30]. When administered prior to or shortly after Ad-VEGF-A^164^, MV and GMP formation were prevented and both angiogenesis and arterio-venogenesis were strikingly inhibited, as judged both by histology and by quantitative analysis using a double tracer method that measured total intravascular plasma volume as well as leaked plasma [28]. However, when given 2 months after Ad-VEGF-A^164^ injection, VEGF Trap had no significant effect on the VM, FA and DV that had by this time become well established.

Together these experiments indicate that “early” and “late” vessels differ substantially, not only in structure but also in their dependence on “exogenous” VEGF-A, and therefore their responsiveness to anti-VEGF therapy (Fig. 1). This does not imply, however, that the endothelial cells of “late” vessels are entirely VEGF-A-independent. Pericytes and smooth muscle cells that are in intimate contact with endothelial cells are known to be a source of VEGF-A and may well be supplying them with adequate VEGF-A for survival. Such a paracellular source of VEGF-A is not likely to be easily inhibited by anti-VEGF-A drugs, especially by large molecules such as Aflibercept that cannot effectively enter the closely apposed smooth muscle cell- or pericyte-endothelial cell interface.

## The path forward

The data presented above indicate that anti-VEGF/VEGFR therapy has serious limitations. Bevacizumab, accompanied by chemotherapy, is able to prolong life marginally in colon cancer and to delay progression modestly in several other cancers. It seems unlikely that these results can be much improved upon with other drugs targeting the VEGF-A/VEGFR axis. However, a distinction needs to be made between anti-VEGF-A/VEGFR and anti-vascular therapy. The results targeting the former should not be interpreted as a negation of the latter. Rather, the point to be made is that there is a need for new targets beyond, or in addition to, the VEGF-A/VEGFR axis. In particular, there is a need to find molecules in or on “late” blood vessels that can serve as new targets. We expect our Ad-VEGF-A^164^ approach to be useful in finding such targets. With it we can develop large numbers of tumor “surrogate” blood vessels of each type at local tissue sites in relatively pure form and at different stages of their progression. This Ad-VEGF-A^164^ approach differs from another useful approach, that of purifying blood vessels and their endothelial cells directly from cancers using SAGE technology [31]. This latter approach requires many hours during which changes in gene expression may take place. Also, the endothelial cells harvested come from blood vessels of all different types, not just from those “late” vessels that may be most fruitful for targeting.

Work in progress is making use of the Ad-VEGF-A^164^ approach to identify genes that are highly expressed in “late” vessels but not in the normal vasculature. Tissues harvested at varying times after Ad-VEGF-A^164^ injection are being harvested and subjected to gene chip and RT-PCR analysis. The hope is to find new potential targets that will allow us to target “late” blood vessels. We appreciate the difficulties that may accompany this approach. At least by histology, “late” vessels such as FA, DV and VM differ less from their normal counterparts than do the “early” vessels such as MV and GMP that have no clearly established normal counterparts. Nonetheless, we have already identified several potential molecular targets that we will be investigating further in the months ahead and hope that one or more of these may be useful therapeutically.

Finally, it should be noted that our Ad-VEGF-A^164^ model might also have a second use. It can provide a valuable tool for screening anti-vascular drugs, as it allows the assessment of drug effectiveness on each type of tumor “surrogate” blood vessel. We suggest that this model can serve as a rapid and relatively inexpensive means for screening the effectiveness of new anti-vascular drugs as they are developed.
